# DST-DETR: Image Dehazing RT-DETR for Safety Helmet Detection in Foggy Weather

**DOI:** 10.3390/s24144628

**Published:** 2024-07-17

**Authors:** Ziyuan Liu, Chunxia Sun, Xiaopeng Wang

**Affiliations:** School of Electronic and Information Engineering, Lanzhou Jiaotong University, Lanzhou 730070, China

**Keywords:** DST-DETR, safety helmet, image dehazing, object detection

## Abstract

In foggy weather, outdoor safety helmet detection often suffers from low visibility and unclear objects, hindering optimal detector performance. Moreover, safety helmets typically appear as small objects at construction sites, prone to occlusion and difficult to distinguish from complex backgrounds, further exacerbating the detection challenge. Therefore, the real-time and precise detection of safety helmet usage among construction personnel, particularly in adverse weather conditions such as foggy weather, poses a significant challenge. To address this issue, this paper proposes the DST-DETR, a framework for foggy weather safety helmet detection. The DST-DETR framework comprises a dehazing module, PAOD-Net, and an object detection module, ST-DETR, for joint dehazing and detection. Initially, foggy images are restored within PAOD-Net, enhancing the AOD-Net model by introducing a novel convolutional module, PfConv, guided by the parameter-free average attention module (PfAAM). This module enables more focused attention on crucial features in lightweight models, therefore enhancing performance. Subsequently, the MS-SSIM + $\ell_{2}$ loss function is employed to bolster the model’s robustness, making it adaptable to scenes with intricate backgrounds and variable fog densities. Next, within the object detection module, the ST-DETR model is designed to address small objects. By refining the RT-DETR model, its capability to detect small objects in low-quality images is enhanced. The core of this approach lies in utilizing the variant ResNet-18 as the backbone to make the network lightweight without sacrificing accuracy, followed by effectively integrating the small-object layer into the improved BiFPN neck structure, resulting in CCFF-BiFPN-P2. Various experiments were conducted to qualitatively and quantitatively compare our method with several state-of-the-art approaches, demonstrating its superiority. The results validate that the DST-DETR algorithm is better suited for foggy safety helmet detection tasks in construction scenarios.

## 1. Introduction

**Figure 1:**
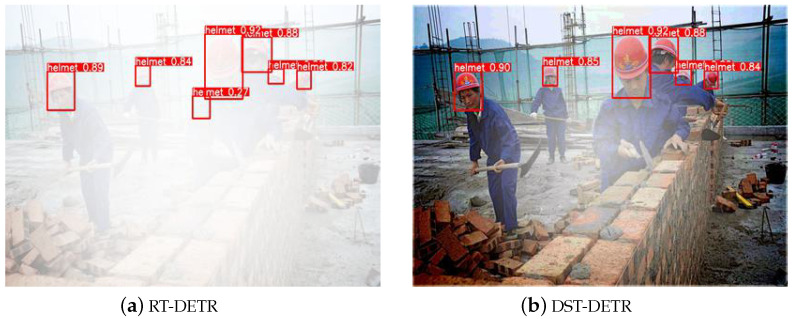
Safety helmet object detection in a foggy environment: DST-DETR (Ours) demonstrates not only better visualization but also higher detection accuracy.

As a crucial piece of personal protective equipment, the safety helmet effectively protects the heads of construction workers from injuries. However, adverse weather conditions can severely impact the accuracy and real-time performance of the data collected by sensors. For instance, varying degrees of image degradation can occur in foggy environments, which significantly affect the performance of helmet detection in construction scenarios. Additionally, risk objects on construction sites often appear as small objects occupying fewer pixels, and the interference from environmental factors on foggy days makes it more challenging to distinguish them from the background or similar objects. Figure 1 illustrates an example of safety helmet object detection in a foggy environment. Using RT-DETR as a benchmark, we compare it with our designed DST-DETR image restoration and detection framework. This preliminary concept suggests that applying dehazing techniques to foggy detection tasks can enhance not only the visual quality of the detected images but also restore latent information. Moreover, optimizing the object detection network with respect to the safety helmets as small objects can significantly enhance the overall accuracy of the detection task.

To tackle the issue of object detection in foggy conditions, Huang et al. [1] developed the DSNet model that utilizes integrated learning for object detection in adverse weather conditions. This model consists of two subnets: a detection subnet and a recovery subnet, responsible for visibility improvement, object categorization, and object positioning. Liu et al. [2] proposed the IA-YOLO framework based on YOLOv3, which adaptively enhances each image at the input under adverse weather conditions, therefore achieving better detection performance. Zhang et al. [3] introduced FINet, a framework that addresses the issue of small sample sizes by creating a synthetic fog dataset. It employs an enhanced YOLOv5 model, incorporating channel attention mechanisms, to achieve superior object detection performance. Li et al. [4] proposed a joint defogging detection framework called BAD-Net, which connects the defogging module and detection module end-to-end. It employs a bilinear branch structure to integrate the features of hazy and defogged images through attention fusion, therefore mitigating the adverse effects of suboptimal defogging module performance to some extent. Although the above methods show good performance on synthetic datasets, the existing dehazing models have slow processing speeds and low robustness, which cannot guarantee real-time dehazing for helmets in construction scenarios. Moreover, the dehazed image will still have some noise, and if the pixels occupied by small objects are too few, the image cannot be accurately recognized after restoration, and the object detection model itself performs poorly in terms of small-object detection accuracy.

To address the limitations of existing object detection algorithms in foggy conditions, this study introduces a fog-specific safety helmet detection approach, DST-DETR. This model combines image dehazing and object detection into a unified framework, enhancing overall system performance under adverse weather conditions. To enhance detailed attention without compromising the dehazing model’s efficiency, this study suggests a novel convolution module, PfConv, to replace the original Conv module in AOD-Net. By combining channel attention and spatial attention and subsequently merging them to emphasize key regions within the feature map, the proposed module aims to streamline the model while capturing intricate details. To address the weak robustness of the dehazing model when dealing with the helmet dataset, the hybrid loss function MS-SSIM + $\ell_{2}$ is utilized to effectively consider detail and texture information during fog map recovery, resulting in better dehazing effects. In the RT-DETR object detection model, the variant ResNet-18 is used as the backbone to reduce the number of parameters. To compensate for its shortcomings in detecting small objects, the neck is improved into a CCFF-BiFPN structure to achieve more efficient feature integration and cross-scale transfer, with the newly added small-object detection layer integrated into the network structure to form the final CCFF-BiFPN-P2 structure. To summarize, the contributions of this paper include the following:

1. Collect 9315 images of actual outdoor construction scenes that include safety helmets, avoiding direct sunlight, and use the atmospheric scattering model to create dehazing and object detection datasets;
2. A new image dehazing model, PAOD-Net, is designed to improve image restoration before the detection module. The model’s performance is improved without increasing its size. Compared with existing methods, it is more effective visually, with improved PSNR and SSIM metrics;
3. The ST-DETR model is proposed for small-object helmet detection, efficiently utilizing shallow features, boosting the semantic depiction of small items, and enhancing helmet detection effectiveness in challenging scenes;
4. Based on the proposed PAOD-Net image dehazing network and ST-DETR object detection network, a new end-to-end real-time helmet detection framework, DST-DETR, is proposed. The model exhibits excellent detection performance in both the foggy helmet detection dataset across various fog densities and in publicly available foggy weather detection datasets.

The organization of the remaining sections of this article is as follows: Section 2 provides an overview of relevant research in image dehazing and object detection. Section 3 describes the creation of dehazing and object detection datasets based on construction scene helmets. Section 4 presents the structure and details of the PAOD-Net model, enhanced for dehazing performance and robustness, and the ST-DETR model, improved for small-object detection. Section 5 expounds upon the details and outcomes of the experiments. Section 6 summarizes the Discussion and Conclusions.

## 2. Related Work

### 2.1. Image Dehazing

Image dehazing algorithms can be categorized into two main types: those that use traditional digital image processing combined with physical models and those based on deep-learning techniques. The first category typically relies on the atmospheric scattering model, with algorithms designed to solve atmospheric light value and transmission matrix to achieve precise results. For instance, He et al. [5] proposed the Dark Channel Prior (DCP) dehaze algorithm. This algorithm estimates haze thickness by identifying the dark channel within the image and recovering a high-quality, haze-free image. In another approach, Zhu et al. [6] adopted the Color Attenuation Prior (CAP) method. This technique involves obtaining the transmittance map and then using the atmospheric scattering model to perform image dehazing. With the advancement of deep learning in image processing, there is growing interest in developing deep-learning-based networks for image dehazing. The pioneering work by Cai et al. [7] introduced deep learning for image dehazing with DehazeNet. This innovative system aims to eliminate fog from images through an end-to-end process, taking a foggy image as input and generating a medium projection map. Subsequently, the fog-free image is reconstructed using atmospheric scattering modeling. However, the original DehazeNet model suffers from limited feature extraction capabilities due to its single-scale, linear convolutional network structure. Ren et al. [8] suggested a dehazing algorithm, Multiscale Convolutional Neural Networks (MSCNN). This method includes a network for coarse-scale and another for fine-scale. Despite improving intricate details of image restoration, it depends on high-quality hardware and cannot quickly complete short-term dehazing. Li et al. [9] introduced the All-in-One Dehazing Network (AOD-Net) dehazing module, which eliminates the need for estimating the transmission matrix and atmospheric light individually. Instead, it produces the dehazed image directly through a compact CNN, allowing seamless integration into the object detection model for optimal compatibility. Qin et al. [10] introduced an attention mechanism into the image dehazing network and proposed the Feature Fusion Attention Network (FFA-Net), which combines both channel and pixel attention, adopts an attention-based feature fusion structure at different levels, and adaptively learns the feature weights from the feature attention module. However, it often suffers from color distortion and contrast degradation.

### 2.2. Object Detection

Object detection, a crucial aspect of visual tasks, has garnered significant attention recently. Contemporary object detection systems fall into two main categories: CNN-based and Transformer-based systems. CNN-based object detection models have been intensively researched in recent years. From initial two-stage detection to single-stage detection, methods based on region proposals combined with CNNs dominate two-stage detection, such as R-CNN [11], Fast R-CNN [12], Faster R-CNN [13], and Mask R-CNN [14]. The most representative single-stage detection models are the YOLO series and SSD [15], with YOLOs being the most widely used, ranging from YOLOv1 to YOLOv10 [16,17,18,19,20,21,22]. With the emergence of Transformer architectures, Carion et al. [23] pioneered integrating Transformers into object detection by introducing DETR, an end-to-end object detection network. DETR harnesses the powerful modeling capabilities of Transformers [24] and the Hungarian matching algorithm, dispensing with manually designed anchors and NMS components found in traditional detection pipelines, thus achieving true end-to-end object detection. Subsequent models like Deformable DETR [25], Conditional DETR [26], and DINO [27] have been proposed to address concerns regarding high computational costs and slow speeds. The advent of RT-DETR [28] has further revolutionized real-time object detection architectures, offering more efficient detection performance suitable for industrial applications.

Traditional helmet detection methods involve manually selecting features. These methods are notably subjective, lack strong generalization capabilities, and face constraints in engineering contexts. As deep-learning algorithms continue to evolve, researchers have gradually applied various object detectors mentioned above to the field of helmet detection, effectively improving the efficiency and accuracy of helmet detection. Zhang et al. [29] enhanced the model’s generalization capability for helmet detection in real-world scenarios by introducing a denoising module and combining channel attention to compress global spatial information. Guo et al. [30] introduced ST-CenterNet, which includes an object-adaptive feature extraction module. This module facilitates bidirectional feature extraction, enhancing the detection accuracy of small helmet objects. Liang et al. [31] proposed a system for detecting helmets using low-altitude remote sensing from UAVs. They introduced a high-precision, attention-weighted fusion network with a single pole that significantly enhances the network’s ability to detect helmets. Song et al. [32] integrated a multi-object tracking algorithm into an object detection network, utilizing Kalman filtering and Hungarian algorithms to predict and track target trajectories in construction scenarios. Experiments proved that the helmet detection speed and accuracy were effectively improved compared to a single detection algorithm and a partial tracking algorithm. Xu et al. [33] integrated a coordinate-space attention module to filter the spatio-temporal data of feature inputs and used multiscale asymmetric convolution to improve the algorithm’s sensitivity to feature scale changes, therefore enhancing helmet detection performance.

## 3. Datasets for Dehazing and Foggy Object Detection

Since there is no public foggy helmet dataset, to ensure the reliability and rationality of the experimental data, we first built a dehazing dataset and an object detection dataset based on construction scene helmets. This experiment collected images of people from outdoor construction scenes. Taking into account the authenticity of foggy scenes, images exposed to direct sunlight were filtered out, resulting in a total of 9315 images. The dehazing dataset and the object detection dataset were then allocated in a ratio of 1:4. A total of 7452 images in the object detection dataset were allocated into training, test, and validation sets in a ratio of 7:2:1. To further improve the model’s robustness, the 1490 images in the object detection test set jointly undertake the testing of dehazing and object detection, serving as a joint test set for the overall framework of the model. The fog synthesis experiment was conducted using the atmospheric scattering model, which has long been recognized as a traditional method for creating hazy images [34]:

(1) $$\begin{array}{c} I(x)=J(x)t(x)+A(1-t(x)) \end{array}$$

where I(x) is the hazy image, and J(x) is the clean image. In addition, *A* denotes the global atmospheric light, and t(x) is the transmission matrix defined as:

(2) $$\begin{array}{c} t\left(x\right)=e^{-\beta d(x)} \end{array}$$

where β is the scattering coefficient of the atmosphere, and d(x) is the distance between the object and the camera defined as:

(3) $$\begin{array}{c} d\left(x\right)=-0.04*\rho +\sqrt{\max(row,col)} \end{array}$$

where ρ is the Euclidean distance from the current pixel to the central pixel, and row and col are the number of rows and columns of the image, respectively.

**Figure 2:**
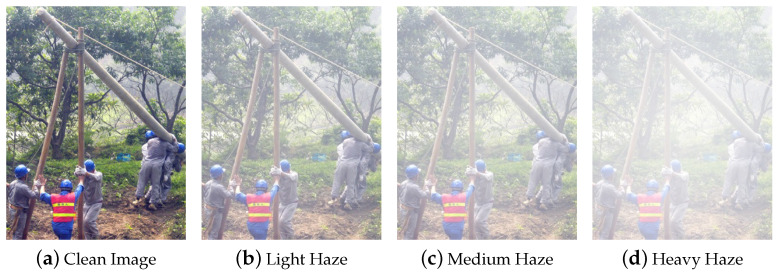
Fog simulation for safety helmet images.

After excluding the object detection dataset, the original dataset retained 1863 images as the training set ground truth for the dehazing dataset, ensuring an approximately 1:1 ratio with the joint dehazing detection test set. The present experiment is founded upon the stochastic utilization of different values of *A* and β in the atmospheric scattering model to generate 15 distinct hazy images for each of the 1863 instances in the dehazing dataset, constituting a total of 27,945 images for the training set. Subsequently, to further evaluate the model’s generalizability, this study, unlike existing foggy condition detection datasets that consist of only one multiple haze levels dataset, created four synthetic sets based on the object detection dataset: Light Haze (A=1,β=0.04), Medium Haze (A=1,β=0.06), Heavy Haze (A=1,β=0.1), and Multiple Haze (where multiple haze levels were mixed in equal proportions). A portion of the generated foggy helmet dataset is illustrated in Figure 2.

## 4. Materials and Methods

With the rapid development of object detection, more researchers are focusing on detection in adverse weather conditions, particularly in foggy environments. To address the severe image degradation caused by the accumulation of fine particulate matter in foggy conditions, some scholars have proposed image enhancement techniques, while others have suggested improvements in the feature extraction stage of detection models. Although these methods have somewhat improved detection performance, they fail to balance visual perception for human eyes and the robustness of detectors. This study, inspired by the concept of “human-machine co-friendliness,” aims to develop a foggy weather object detection framework that meets human visual needs and excels in detecting small objects with robust performance.

**Figure 3:**
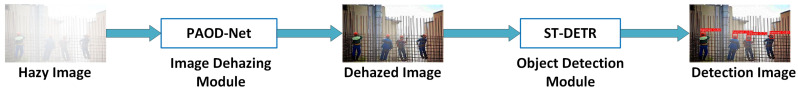
DST-DETR network structure diagram: PAOD-Net for image dehazing module, and ST-DETR for object detection module.

We propose an end-to-end dehazing helmet monitoring system that implements a helmet detection workflow driven by image restoration in foggy scenarios, as shown in Figure 3. The comprehensive workflow consists of two main components: the image dehazing module and the object detection module. The former performs image restoration under various degrees of foggy conditions, while the latter detects whether construction workers are wearing helmets.

### 4.1. PAOD-Net Image Dehazing Module

Currently, image dehazing models are reaching new levels of performance on public datasets. However, their image restoration capabilities are often lacking in specific foggy scenarios, particularly under heavy haze conditions, where they significantly impact detector performance. Since the goal is to enable detectors to achieve outstanding detection performance, the primary consideration for image dehazing models should be dehazing efficiency—keeping the model lightweight while ensuring rapid dehazing to better integrate with object detection models.

**Figure 4:**
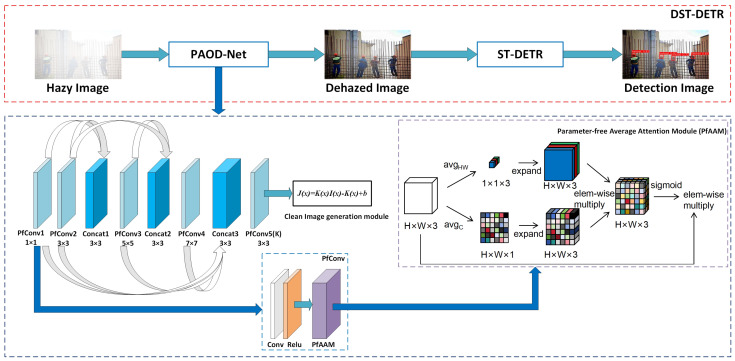
PAOD-Net network structure diagram in the DST-DETR framework.

Therefore, we conducted experiments using AOD-Net, the most lightweight image dehazing model available, as our benchmark. However, lightweight dehazing models typically lack robustness. To address this, we improved the model’s performance while maintaining its lightweight nature by proposing a new PfConv module. This module introduces a parameter-free average attention module (PfAAM), which balances both spatial and channel attention. Finally, to overcome the limitations of the $\ell_{2}$ loss function, which focuses only on pixel-level differences and disregards human visual perception, we adopted a mixed loss function, MS-SSIM + $\ell_{2}$. This approach improves the model’s restoration capabilities by emphasizing perceptual changes in the image, resulting in the final PAOD-Net model, as shown in Figure 4.

#### 4.1.1. End-to-End Dehazing Network Design

PAOD-Net extends the end-to-end network framework design of AOD-Net, resulting in a lightweight network capable of rapid dehazing. This end-to-end network structure enables PAOD-Net to be seamlessly integrated into object detection models. Unlike most models that require separate estimation of the transmission matrix and atmospheric light, PAOD-Net reformulates Equation (1) based on the revised atmospheric scattering model as follows:

(4) $$\begin{array}{cc} J(x) & =K(x)I(x)-K(x)+b,where\\ K(x) & =\frac{\frac{1}{t(x)}\left(I\left(x\right)-A\right)+\left(A-b\right)}{I(x)-1} \end{array}$$

where *b* is a constant bias with a default value set to 1. The core idea is to integrate the two parameters t(x) and *A* from the atmospheric scattering model into K(x), which in turn directly minimizes the pixel-domain reconstruction error. Since K(x) depends on I(x), the objective is to construct an input adaptive depth model with parameters that change according to the input foggy sky image to reduce the reconstruction error between the output J(x) and the true image. The PAOD-Net network obtains a clean image in two steps, first by going through the K-estimation module, which is defined by the input I(x) Estimation of K(x) and then using K(x) as an input adaptive parameter to estimate J(x), which in turn results in a clean image.

A fundamental element of PAOD-Net is the K-estimation module, which is essential for determining both the depth and the relative concentration of haze. To maintain a lightweight model, only five PfConv modules are used. By integrating filters of various sizes to form multiscale features, the intermediate layers of coarse-scale and fine-scale network features are connected. This multiscale design captures features at different scales, with intermediate connections partially compensating for information loss during the convolution process. After passing through the K-estimation module, the clean image generation module, which consists of an element-wise multiplication layer and several element-wise addition layers, produces the restored image using Equation (4).

#### 4.1.2. Parameter-Free Average Attention Module

With the rapid development of attention mechanisms, an increasing number of these mechanisms are being introduced into the field of image dehazing. However, most attention mechanisms require parameterized upscaling and downscaling operations. Since the convolution modules used in PAOD-Net have an output channel number of only three, such operations can easily lead to the loss of important information in small-channel modules. Therefore, we introduce PfAAM, a parameter-free attention module that maintains consistent input and output dimensions.

Figure 4 demonstrates the overall structure and computation of PfAAM [35]. To tailor to the specific network structure, the input feature map shape is optimally adjusted from H×W×C to H×W×3, wherein $F\in R^{H\times W\times 3}$ serves as the intermediary input. PfAAM segregates the inputs into two attention parts, computing the spatial attention component $A_{sp}\in R^{H\times W\times 1}$ by averaging spatial features along channels and the channel attention component $A_{ch}\in R^{1\times 1\times 3}$ by averaging features along the spatial dimension of the feature map. Subsequently, the resulting attention maps are elongated along their respective diminishing dimensions and reorganized to emphasize crucial aspects of the input feature map. The rearranged attention map then employs a sigmoid gating mechanism to enhance the representation of the input. The entire process can be summarized as follows:

(5) $$\begin{array}{c} A_{sp}\left(x_{H\times W}\right)=\frac{1}{3}\sum_{i=1}^{3}x_{H\times W}\left(i\right) \end{array}$$

(6) $$\begin{array}{c} A_{ch}\left(y_{3}\right)=\frac{1}{H\times W}\sum_{i=1}^{H}\sum_{j=1}^{W}y_{3}\left(i,j\right) \end{array}$$

(7) $$\begin{array}{c} F^{\prime }=\sigma \left(A_{sp}⊗A_{ch}\right)⊗F \end{array}$$

where $x_{H\times W}$ represents the average of each spatial element, $y_{3}$ represents the average along its spatial dimension, ⊗ is the element-wise multiplication, σ is the sigmoid function, *F* is the input of the feature map, and $F^{\prime }$ is the output of PfAAM. Unlike attentional modules, which learn parameters to highlight features, PfAAM is parameter-free and instead focuses on features solely through spatial and channel-wise averaging.

#### 4.1.3. Mixed Loss Function

In the realm of image dehazing, the loss function measures the difference between the dehazed image and the true haze-free image, therefore guiding the optimization and learning trajectory of the model during training. Due to its straightforwardness and convex nature, $\ell_{2}$ is typically favored as the loss function for image dehazing tasks. In contrast to $\ell_{1}$, $\ell_{2}$ employs the summation of the squares of the differences in pixel values between the dehazed image and the true haze-free image, penalizing significant discrepancies while being more lenient towards minor discrepancies. While this method adeptly addresses the issue of noise, it disregards structural details, occasionally resulting in noticeable speckle-like artifacts in the restored image. The $\ell_{2}$ loss function can be articulated as follows:

(8) $$\begin{array}{c} L^{\ell_{2}}\left(P\right)=\frac{1}{N}\sum_{p\in P}{\left(x\left(p\right)-y\left(p\right)\right)}^{2} \end{array}$$

Compared to the original AOD-Net architecture, which solely employs the $\ell_{2}$ loss function, PAOD-Net integrates a hybrid MS-SSIM + $\ell_{2}$ loss function. Given that image dehazing aims to produce visually coherent and aesthetically pleasing results, metrics imbued with perceptual motivation, such as SSIM, warrant exploration. SSIM operates on a perceptual framework, conceptualizing image degradation as a perceptual alteration of structural information. It prioritizes critical perceptual phenomena, including luminance, contrast, and texture structure, which are evaluated based on the pixel *p*, calculated using the following formula:

(9) $$\begin{array}{cc} \mathrm{SSIM}(p) & =\frac{2\mu_{x}\mu_{y}+C_{1}}{\mu_{x}^{2}+\mu_{y}^{2}+C_{1}}\cdot \frac{2\sigma_{xy}+C_{2}}{\sigma_{x}^{2}+\sigma_{y}^{2}+C_{2}}\\ \; & =l(p)\cdot cs(p) \end{array}$$

where both the means and deviations do not depend on the pixel *p*. Both the means and the standard deviations are calculated using a $G_{\sigma_{G}}$ Gaussian filter, where the standard deviation is $\sigma_{G}$. $C_{1}$ and $C_{2}$ are constants that prevent the denominator from being zero. l(p) and cs(p) gauge the luminance comparison and the composite structural similarity comparison between *x* and *y* at pixel *p*, respectively. The SSIM loss function can be defined as follows:

(10) $$\begin{array}{c} L^{\mathrm{SSIM}}\left(P\right)=\frac{1}{N}\sum_{p\in P}1-\mathrm{SSIM}\left(p\right) \end{array}$$

However, the convolutional nature of our network allows for further rewriting of the loss function:

(11) $$\begin{array}{c} L^{\mathrm{SSIM}}\left(P\right)=1-\mathrm{SSIM}\left(\tilde{p}\right) \end{array}$$

where $\tilde{p}$ is the center pixel of patch *P*. It is preferred to use a multiscale version of SSIM, i.e., MS-SSIM, rather than fine-tuning $\sigma_{G}$. Given a binary pyramid of *M* layers, MS-SSIM can be defined as follows:

(12) $$\begin{array}{c} \mathrm{MS}-\mathrm{SSIM}\left(p\right)=l_{M}^{\alpha }\left(p\right)\cdot \prod_{j=1}^{M}cs_{j}^{\beta_{j}}\left(p\right) \end{array}$$

where $l_{M}$ and $cs_{j}$ represent the terms defined in Equation (9) at scales *M* and *j*, respectively. For simplicity, we set $\alpha =\beta_{j}=1$ for j={1,…,M}. Analogous to Equation (11), we can approximate the loss for patch *P* with the loss computed at its central pixel $\tilde{p}$:

(13) $$\begin{array}{c} L^{\mathrm{MS}-\mathrm{SSIM}}\left(P\right)=1-\mathrm{MS}-\mathrm{SSIM}\left(\tilde{p}\right) \end{array}$$

The PAOD-Net model uses a combination of MS-SSIM and $\ell_{2}$ weighting as the loss function:

(14) $$\begin{array}{c} L^{\mathrm{Mix}}=\alpha \cdot L^{\mathrm{MS}-\mathrm{SSIM}}+\left(1-\alpha \right)\cdot G_{\sigma_{G}^{M}}\cdot L^{\ell_{2}} \end{array}$$

where $\sigma_{G}^{i}=\left\{0.5,1,2,4,8\right\}$. To accelerate the training process, we choose to use *M* distinct values for $\sigma_{G}$ on full-resolution patches rather than computing the *M* levels of the pyramid *P*, with each value being twice the previous one.

### 4.2. ST-DETR Object Detection Module

Current object detectors are showing increasingly better performance on public datasets like COCO [36]. However, in specialized fields such as safety helmet detection, benchmark models do not necessarily exhibit optimal detection performance. Safety helmets often appear as small objects on construction sites and are frequently subject to occlusion and background interference, making most detectors inadequate for the task. Therefore, it is necessary to use a detector specifically designed for small safety helmet objects and improve it, unlocking the full potential of its foundational framework.

We chose the Real-Time DEtection TRansformer (RT-DETR) as our foundational framework. RT-DETR is a real-time, end-to-end object detector that leverages the efficiency of a Vision Transformer (ViT) to handle multiscale features adeptly, delivering real-time performance while maintaining high precision. RT-DETR employs a CNN architecture for its backbone network, utilizing the internally developed HGNet by Baidu. The encoding module of RT-DETR incorporates a highly efficient hybrid encoder that addresses multiscale features by separating internal scale interactions and merging cross-scale elements. This unique ViT framework minimizes computational costs, facilitating real-time object detection. Meanwhile, the decoding segment of RT-DETR employs a multi-layer Transformer decoder, permitting flexible layer selection during inference. This approach adaptively adjusts inference speed without the need for retraining.

**Figure 5:**
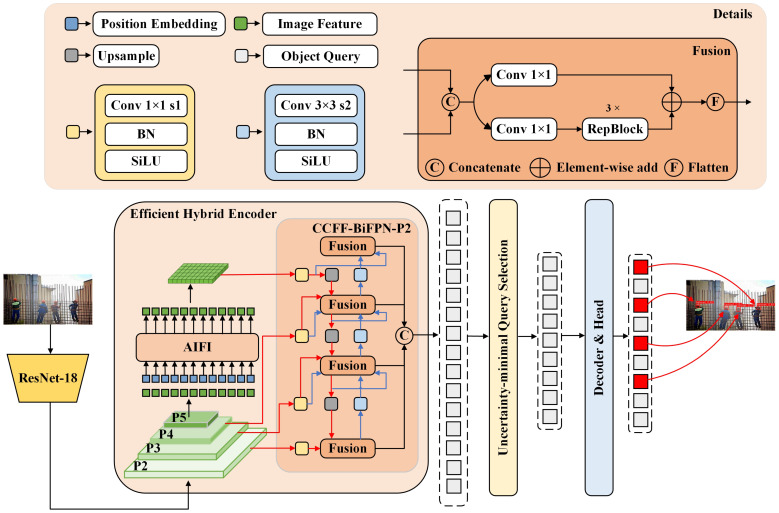
ST-DETR network structure diagram.

Our ST-DETR is a detector specifically designed for small-object detection in safety helmets. We utilized the variant ResNet-18 as the backbone of RT-DETR to lighten the model while preserving its accuracy. Inspired by the effectiveness of BiFPN in handling small objects [37] and acknowledging RT-DETR’s limited detection ability for such objects, we substituted the entire neck structure with a CCFF-BiFPN configuration. Additionally, we introduced a small-object detection layer on top of this structure to further enhance the feature extraction capabilities for small objects, resulting in an improved network termed CCFF-BiFPN-P2. The architecture of our ST-DETR model is depicted in Figure 5.

#### 4.2.1. Variant ResNet Architecture

**Figure 6:**
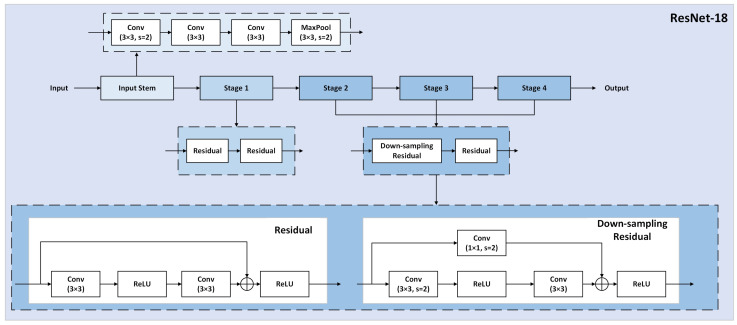
The architecture of the variant ResNet-18.

In recent years, various lightweight network architectures such as VGGNet, ResNet, and MobileNet have been proposed. ResNet [38] networks are commonly employed in object detection and recognition due to their straightforward design and effectiveness. The key characteristic of ResNet networks is their residual block structure, which enhances the depth of the convolutional neural network and resolves issues related to vanishing gradients or gradient explosions. Due to the profound impact of ResNet, subsequent research has produced various ResNet variants. This study partitions the variant ResNet-18 model into a single input stem and four subsequent stages. It serves as the backbone for feature extraction in RT-DETR, streamlining the model’s structure and minimizing unnecessary computations. Three 3 × 3 convolutional layers have been used to replace the input stem, which was previously a single 7 × 7 convolutional layer. Through experimentation, it has been observed that the computational cost of convolution scales quadratically with the width or height of the kernel. Specifically, the computational expense of a 7 × 7 convolution is 5.4 times higher than that of a 3 × 3 convolution [39]. Therefore, substituting a single 7 × 7 convolution with three traditional 3 × 3 convolutions proves to be beneficial. In this replacement, the first two convolutions have an output channel size of 32, while the third convolution has an output channel size of 64. Each of the four stages is composed of two residual blocks with channel sizes of 64, 128, 256, and 512, respectively. The connections between the residual blocks are termed “network shortcuts,” which can skip one or multiple layers, facilitating the transmission of network information to deeper layers. These connections are classified into residual blocks and downsampling residual blocks. In particular, downsampling residual blocks achieve dimensionality reduction and channel matching through the utilization of 1 × 1 convolutions. The architecture of the variant ResNet-18 is shown in Figure 6.

#### 4.2.2. Feature Extraction Network

The neck network of RT-DETR referred to as the Efficient Hybrid Encoder, comprises two components: Attention-based Intra-scale Feature Interaction (AIFI) and CNN-based Cross-scale Feature Fusion (CCFF), as depicted in Figure 5. The AIFI module exclusively processes the feature map of layer P5. Compared to previous DETR models that operate on multiscale features, this approach significantly reduces computational overhead and improves processing speed without noticeably compromising model performance.

**Figure 7:**
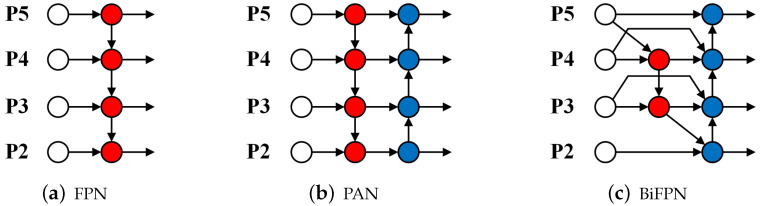
Feature extraction network design: (**a**) FPN, (**b**) PAN, and (**c**) BiFPN.

Regarding the CCFF, from the perspective of YOLO, this structure can be interpreted as either an FPN or PAN architecture, as depicted in Figure 7a,b. The FPN structure effectively transmits deep feature data to shallower layers, enhancing their understanding of critical and high-level information. Meanwhile, the PAN structure aids in accurately positioning information transitioning from shallower layers to deeper layers with more abundant features, therefore significantly improving the model’s capacity to detect subtle features. However, when dealing with small objects, BiFPN [40] demonstrates superior performance, as depicted in Figure 7c. First, it introduces an additional pathway from high to low resolution, enhancing the efficiency of feature fusion compared to FPN structures. Second, it eliminates nodes that receive input solely from a single node, making BiFPN lighter and more efficient than PAN. The BiFPN utilizes skip connections to create pathways from the input layer to the output layer, enabling the neural network to understand the harmony between low-level and high-level characteristics while merging features. To compensate for inadvertently filtered typical feature information in the RT-DETR backbone, we innovatively integrated BiFPN into our model. These innovative pathways skillfully preserve and integrate the initial features extracted directly from the backbone network into the detection feature map.

#### 4.2.3. Small-Object Detection Layer

Small-object detection is often considered one of the most challenging tasks in deep-learning object detection. In practical construction scenarios, such as those involving safety helmets, small objects are commonly encountered due to factors like distance and occlusion. However, when small objects pass through downsampling feature layers and numerous deep convolutions, a significant amount of shallow positional information can be lost. Coupled with the limited number of pixels in small objects, this can lead to issues such as missed detections and false positives. In this context, we strategically improved the neck structure of RT-DETR to optimize it specifically for small-object detection.

As shown in Figure 5, we augmented the existing RT-DETR model with a new detection layer, accompanied by an additional detection head specifically designed for the recognition of small objects. The new detection head processes images at higher resolutions in the shallow network layers, capturing more detailed information about small objects, even in low-resolution images. By generating more feature points for objects with lower pixel values in high-resolution images, the recognition performance of small objects is greatly improved. Additionally, the original deep detection head still handles large objects within the network structure. This new design effectively enhances the recognition capability of small objects while maintaining computational efficiency.

By implementing this enhancement approach, we have not only reinforced the integration of positional and feature data within the model but also greatly improved the accuracy in identifying minute objects. Experimental verification has shown that this enhancement approach significantly benefits the detection of small safety helmets, therefore enhancing the accuracy and robustness of the model in practical construction scenarios.

## 5. Results

### 5.1. Experimental Setup

To attain rapid and reliable outcomes, the entire methodology is executed on a single workstation equipped with an NVIDIA GeForce RTX 4090 GPU, an Intel i7 CPU, and 32 GB of RAM. All coding endeavors are based on CUDA 11.8, PyTorch 2.0.1, and Python 3.9. During the training process for object detection, the batch size is uniformly set to 4, the number of workers is set to 4, image input dimensions are configured to 640 × 640 pixels, the final learning rate is set to 0.0001, momentum is set to 0.9, and the number of training epochs is set to 200. The training parameters for all dehazing models are configured to their optimal performance values, with specifics on our dehazing model parameters to be detailed in subsequent experimental sections.

### 5.2. Evaluation Indicators

Structural similarity (SSIM) and peak signal-to-noise ratio (PSNR) are generally used to measure the quality of images restored by dehazing algorithms.

PSNR is evaluated by calculating the mean square error (MSE) between the clean image and the restored image. The smaller the value of MSE, the larger the value of PSNR, indicating a better dehazing effect of the model. For M×N-sized images, MSE and PSNR can be obtained by Equations (15) and (16), respectively:

(15) $$\begin{array}{c} \mathrm{MSE}=\frac{1}{M\times N}\sum_{i=0}^{M-1}\sum_{j=0}^{N-1}{\left[X\left(i,j\right)-Y\left(i,j\right)\right]}^{2} \end{array}$$

(16) $$\begin{array}{c} \mathrm{PSNR}=10\cdot \log_{10}\left(\frac{{\mathrm{MAX}}_{X}^{2}}{\mathrm{MSE}}\right) \end{array}$$

where ${\mathrm{MAX}}_{X}^{2}$ is the maximum possible pixel value of the clean image.

SSIM is distinct from Equation (9) in that it measures the similarity between two images by evaluating their luminance, contrast, and structure. The expression for SSIM is as follows:

(17) $$\begin{array}{cc} \mathrm{SSIM}(X,Y) & =l(X,Y)\cdot c(X,Y)\cdot s(X,Y)\\ \; & =\frac{2\mu_{X}\mu_{Y}+C_{1}}{\mu_{X}^{2}+\mu_{Y}^{2}+C_{1}}\cdot \frac{2\sigma_{X}\sigma_{Y}+C_{2}}{\sigma_{X}^{2}+\sigma_{Y}^{2}+C_{2}}\cdot \frac{\sigma_{XY}+C_{3}}{\sigma_{X}\sigma_{Y}+C_{3}} \end{array}$$

where $\sigma_{X}$ and $\sigma_{Y}$ denote the variance of images *X* and *Y*; $\mu_{X}$ and $\mu_{Y}$ denote the mean of images *X* and *Y*; $C_{1}$, $C_{2}$, and $C_{3}$ are constant terms; $\sigma_{XY}$ denotes the covariance of images *X* and *Y*.

In the object detection task, mean average precision (mAP), frames per second (FPS), and the total number of parameters (Params) serve as metrics for evaluating both the algorithm’s detection accuracy and speed, as well as the model’s size. mAP is the mean of the average precision (AP) for each type of object and is calculated as follows:

(18) $$\begin{array}{c} \mathrm{mAP}=\frac{1}{C}\sum_{i=0}^{C}{\mathrm{AP}}_{i} \end{array}$$

where *C* denotes the total number of categories, and ${\mathrm{AP}}_{i}$ represents the AP for the class numbered *i*. AP is calculated using the interpolation method as follows:

(19) $$\begin{array}{c} \mathrm{AP}=\int_{0}^{1}P\left(R\right)dR \end{array}$$

where P(R) is the mapping relationship between precision (P) and recall (R). Precision and recall are also prevalent evaluation metrics in object detection, and their computation methods are as follows:

(20) $$\begin{array}{c} P=\frac{\mathrm{TP}}{\mathrm{TP}+\mathrm{FP}} \end{array}$$

(21) $$\begin{array}{c} R=\frac{\mathrm{TP}}{\mathrm{TP}+\mathrm{FN}} \end{array}$$

where TP denotes the number of objects predicted as positive samples that are actually positive; FP signifies the number of objects predicted as positive samples that are actually negative; FN represents the instances predicted as negative samples that are actually positive.

### 5.3. Image Dehazing Experiments

**Figure 8:**
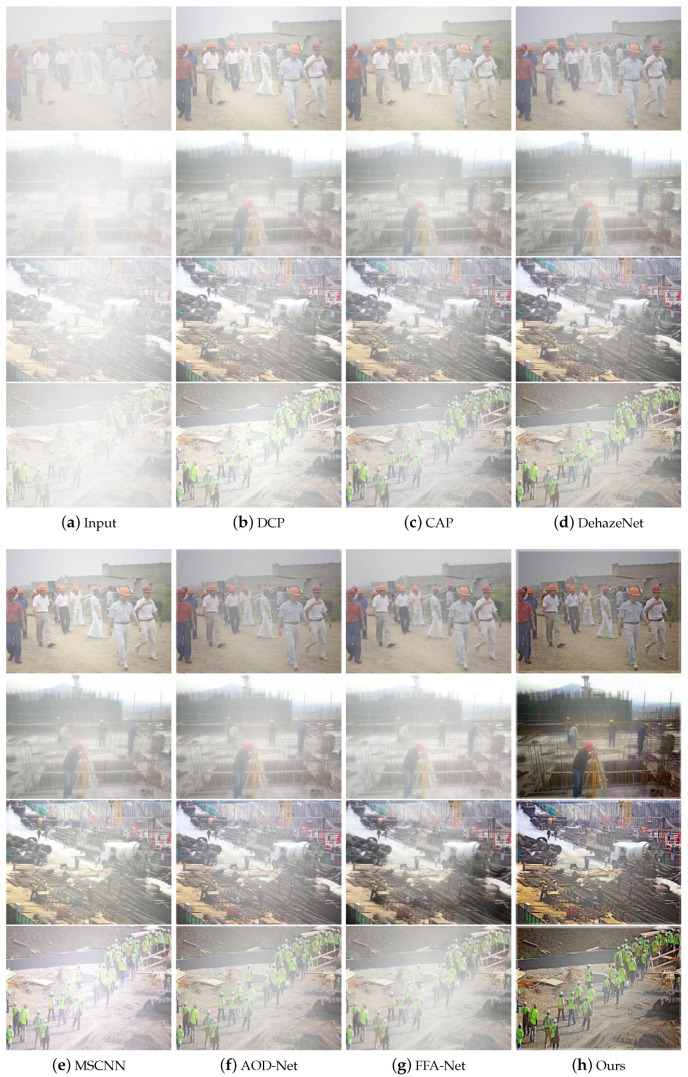
Qualitative comparison of different dehazing methods under heavy haze.

The quality of image recovery after dehazing can greatly affect the accuracy of object detection in foggy environments. In this paper, we design PAOD-Net (Ours) to compare with traditional DCP and CAP algorithms and deep convolutional neural network-based algorithms, including DehazeNet, MSCNN, AOD-Net, and FFA-Net. To better serve the object detection network, this paper selects the heavy haze test set, which has the most significant impact on the network, for a dehazing effect comparison. As illustrated in Figure 8, images from various angles and backgrounds are chosen to verify the algorithm’s robustness. From the figure, it can be seen that the aforementioned dehazing algorithms generally exhibit uneven dehazing, resulting in darker, less clear images, which significantly impact subsequent image recognition tasks. As illustrated in Figure 8h, our algorithm restores more details, markedly enhances image quality, and improves both saturation and color, rendering the visual effect much clearer. We hypothesize that the dehazing network makes the helmet colors more vivid, increasing the detector’s sensitivity to the helmet’s color features. This distinction helps differentiate the helmet from similarly colored background objects, further addressing issues of missed and false detections.

To make a full range of accurate comparisons, we evaluated them on three test sets with different haze concentrations. To evaluate the effectiveness and real-time performance of the aforementioned haze removal algorithms, the objective evaluation metrics PSNR and SSIM, as well as the average running time of each model, were employed. The average objective evaluation results of dehazed images for test sets with various haze densities, as well as the average running times of different models, are shown in Table 1. As shown in the table, for the objective metrics PSNR and SSIM, while DCP achieves faster dehazing speed, its dehazing effect is suboptimal across all test sets. CAP and MSCNN perform well in light haze but poorly in heavy haze. DehazeNet and FFA-Net show excellent dehazing effects in light and medium haze, but their performance in heavy haze is unsatisfactory, and their dehazing times are too long to meet real-time requirements. Although AOD-Net has a very fast dehazing speed, meeting real-time requirements, its overall dehazing effect is relatively poor. Comparatively, the proposed PAOD-Net method demonstrates the strongest overall performance across all haze densities, achieving the best dehazing effect for heavy haze and leading performance for light and medium haze. Additionally, it significantly outperforms the aforementioned algorithms in dehazing efficiency. Compared to AOD-Net, the dehazing efficiency remains unaffected, and robustness is greatly enhanced, which is the primary reason for using PAOD-Net in this experiment.

To further demonstrate the effectiveness of this experiment for improving AOD-Net, ablation experiments were performed on PAOD-Net. The design compares the impact on model performance of replacing the PfConv module and various combinations of loss functions, along with the experimental results on the multiple haze level test set, as shown in Table 2. For MS-SSIM, the Gaussian filters were constructed by setting $\sigma_{G}^{i}=\left\{0.5,1,2,4,8\right\}$. The loss function for MS-SSIM + $\ell_{1}$ used α=0.025, and MS-SSIM + $\ell_{2}$ used α=0.1, following [41]. As shown in the table, the PfConv module significantly enhances both the PSNR and SSIM of the model. When using the $\ell_{2}$ loss function alone, the SSIM value is lower than when using the $\ell_{1}$ loss function alone, and the combination of MS-SSIM and $\ell_{2}$ loss function yields the best performance. The experiments demonstrate that our proposed improvements in PAOD-Net are highly effective, greatly enhancing dehazing effects and better restoring image quality. The effectiveness of our model is attributed to the PfConv module we designed, which, compared to the original Conv module, focuses more on critical features. This mechanism efficiently combines different features. Additionally, the introduction of the MS-SSIM + $\ell_{2}$ loss function has profoundly impacted the robustness of image restoration.

### 5.4. Object Detection Experiments

From Figure 8, it can be seen that the helmet images, after dehazing, display more distinct contours, enhanced information richness, and improved recognizability and contrast. Therefore, we combined the dehazing model with the object detection model for joint training, enabling effective helmet detection in foggy conditions. To assess the effectiveness of the joint optimization model introduced in this study for detecting helmets in hazy conditions, this experiment was conducted on the joint test set for both horizontal and vertical comparison experiments. The improved ST-DETR (Ours) algorithm is compared with the current most popular Faster R-CNN, SSD, YOLO series, and RT-DETR object detectors after PAOD-Net dehazing experiments. To ensure that the model sizes are similar, Faster R-CNN under ResNet50, SSD under VGG16, and YOLOv5-M, YOLOv8-M, YOLOv9-M, YOLOv10-M, and RT-DETR-R18 are selected as the baseline models for the experiments, and the mAP detection results of the different models under different haze concentrations are given in Table 3. From the table, it can be seen that the YOLO and DETR series exhibit leading detection performance, with each YOLO model demonstrating considerable competitiveness. However, compared to our model, nearly all models display a common issue: they perform well in light and medium haze but poorly in heavy haze, lacking good generalization capabilities to handle complex and variable adverse weather. In comparison to RT-DETR, our model shows improvements in mAP@0.5 by 3.7%, 3.6%, and 4.0% under light, medium, and heavy haze conditions, respectively. This demonstrates that, supported by a high-level dehazing model, our enhanced object detection model is suitable for any haze density and performs best in heavy haze. This endows the entire model framework with exceptional dehazing detection performance and robustness.

In addition to verifying accuracy, the model’s overall evaluation should also be competitive. Table 4 provides the detection results of different detectors under various haze concentrations. From the table, it can be seen that our model outperformed the pre-improved RT-DETR-R18, with an mAP@0.5 increase of 4.7% and an mAP@0.5:0.95 enhancement of 8.4%. Compared to RT-DETR-L, our model shows an mAP@0.5 rise of 4.5% and an mAP@0.5:0.95 increase of 7.4%. These results indicate that our optimizations for helmet detection can achieve high precision. Furthermore, our detection accuracy surpasses that of YOLOv5-M, YOLOv8-M, YOLOv9-M, and YOLOv10-M, demonstrating that our model can attain superior accuracy with similar parameter quantities while also meeting real-time requirements. In comparison to YOLOv7, our model not only achieves higher precision but also features fewer parameters, showcasing its lightweight nature. This signifies that our model can deliver high-precision performance while emphasizing its lightweight characteristics, making it more suitable for deployment in scenarios requiring helmet detection, such as drones and surveillance cameras.

**Figure 9:**
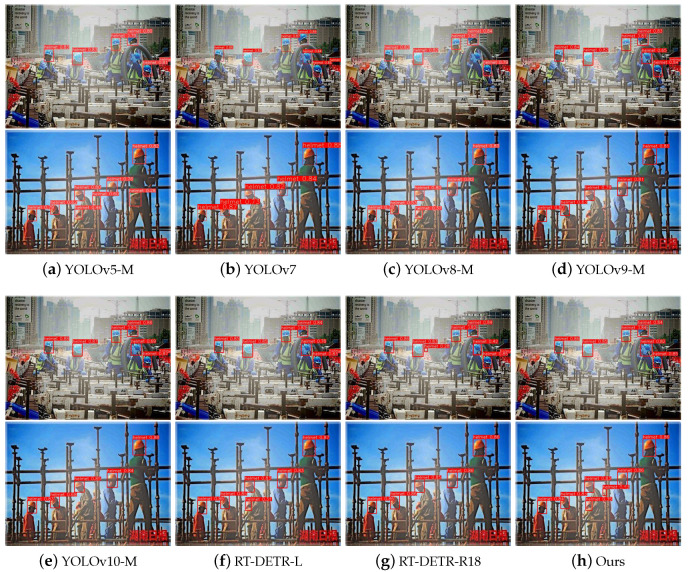
Qualitative comparison of different object detection models.

To more intuitively validate the object detection effectiveness and robustness of the proposed ST-DETR model, we present the visualization results of ST-DETR alongside YOLOv5-M, YOLOv7, YOLOv8-M, YOLOv9-M, YOLOv10-M, RT-DETR-L, and RT-DETR-R18 in Figure 9 for an extensive qualitative comparison. The figure illustrates the multi-object detection performance of various detectors for helmets in different backgrounds, angles, and colors.

From the comparison of the two rows in the figure, it is evident that for safety helmet detection, which often appears as small objects in construction scenes, our method not only identifies helmets heavily obscured by other objects to address missed detection but also prevents helmets from being misidentified due to background objects of similar color. The effectiveness of our method is attributed to embedding the small-object layer into the BiFPN structure, which is inherently favorable to small-object detection, and further optimizing for high-precision helmet detection.

To further validate the effectiveness of this experiment for the improvement of the RT-DETR model, ablation experiments were conducted on ST-DETR. The design uses ResNet-18 as the benchmark, comparing the effect of adding BiFPN and combining a small target layer on the detector’s performance, as shown in Table 5. The table demonstrates that under conditions of multiple haze, our proposed ST-DETR model significantly benefits from the BiFPN structure. The introduction of P2 has had an embellishing effect, effectively enhancing the detection performance of RT-DETR. Consequently, the model’s robustness is greatly improved, enabling it to effectively counteract the impact of adverse environments.

To verify the effectiveness of the overall framework of joint image dehazing and object detection, the dehazing model and the object detection model before and after improvement were subjected to full ablation experiments in the multiple haze test set, as shown in Table 6. The table demonstrates that as the degree of image restoration improves—from no dehazing to dehazing with the AOD-Net model to dehazing with our designed PAOD-Net model—the mAP@0.5 values of the object detection model correspondingly increase. Notably, the improved ST-DETR model exhibits enhancements of 0.107, 0.224, and 0.427 compared to RT-DETR. These results indicate that better image restoration significantly enhances the performance of the object detection model, further underscoring the indispensable roles of the PAOD-Net image dehazing model and the ST-DETR object detection model within our overall dehazing and object detection framework.

Finally, to verify whether the proposed DST-DETR dehazing detection framework is applicable to other foggy detection tasks, we compared it against public datasets. We selected public datasets from both real and simulated perspectives for different scenarios. One dataset is the real foggy dataset RTTS from RESIDE-β [42], which consists of 4322 real-world hazy images collected from the Internet, primarily covering traffic and driving scenes. The other is the simulated foggy dataset SFID [3], containing 13,718 insulator images. The performance of various foggy weather detection models on public datasets was quantitatively evaluated as depicted in Table 7.

From Table 7, it can be observed that on the RTTS dataset, the foggy weather detection performance of DSNet, IA-YOLO, and BAD-Net is inferior to that of the original RT-DETR model used in this experiment. Our proposed DST-DETR foggy weather detection framework, based on RT-DETR, improved the Precision, Recall, mAP@0.5, and mAP@0.5:0.95 metrics by 3.1%, 7.1%, 6.8%, and 3.6%, respectively. Additionally, on the SFID dataset, our DST-DETR improved the mAP@0.5:0.95 metric by 4.1% compared to RT-DETR, with the remaining metrics showing no significant change due to their proximity to 1. Compared to the original paper’s FINet, our model also achieved advantages in Recall and mAP@0.5, while Precision and mAP@0.5:0.95 showed no significant disadvantages. Based on the above quantitative analysis, it is evident that our proposed DST-DETR foggy weather detection model not only excels in detecting safety helmets in foggy conditions but also possesses strong generalizability, demonstrating commendable performance on public datasets and is easily adaptable to other foggy weather detection tasks.

**Figure 10:**
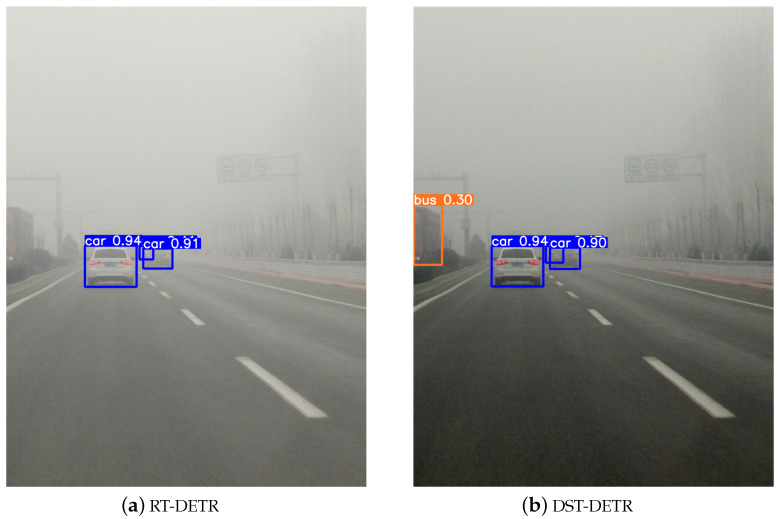
Qualitative comparison of the proposed model and the original model on the RTTS dataset.

**Figure 11:**
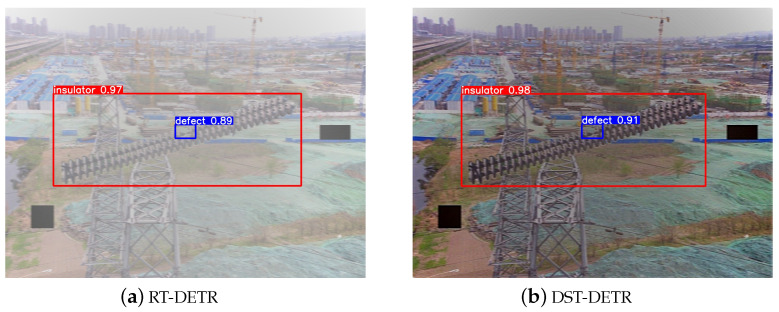
Qualitative comparison of the proposed model and the original model on the SFID dataset.

Subsequently, through experiments, the degree of image restoration and the detector’s detection performance were analyzed from a visual perception perspective. The visualization results of the proposed model compared to the original model are shown in Figure 10 and Figure 11 for qualitative comparison.

The comparison between Figure 10a,b shows that our designed DST-DETR framework better identifies buses, avoiding missed detections and achieving a degree of image restoration. It not only defogs but also enhances image clarity, as seen in the deblurred traffic sign in the upper right corner of the image. The comparison between Figure 11a,b demonstrates that our DST-DETR framework exhibits superior detection performance for both categories. These comparative advantages are attributed to our enhancements in the image restoration capability of the dehazing model and the improvements made to the detector.

Notably, although the DST-DETR framework achieved excellent detection performance on both real and simulated foggy datasets, the comparison between Figure 10b and Figure 11b reveals that its image restoration effect is superior on the simulated dataset. This is evident in the richer color features and details. The reason for this is that the simulated dataset includes a dedicated dehazing dataset, which contains a synthetic fog training set for each clean image used in dehazing experiments. In conclusion, the DST-DETR framework can achieve excellent dehazing detection results on both real and simulated foggy datasets, making it meaningful to further deploy and implement it in real-world scenarios.

## 6. Discussion and Conclusions

To address the requirements of helmet detection in real-world construction scenarios with fog, this paper proposes a dehazing-driven helmet detection framework based on the RT-DETR model, named DST-DETR. This framework consists of the dehazing model PAOD-Net and the object detection model ST-DETR to achieve combined dehazing and detection. By conducting both independent and joint experiments, we demonstrate the efficacy and performance benefits of our proposed framework.

To address the lack of helmet datasets in foggy scenarios, we created dehazing detection datasets with various haze densities using an atmospheric scattering model and established a combined test set to enhance the rationality and authenticity of the experiments. To solve the problem of balancing the image restoration capability of the dehazing model with detection speed and to maximize the performance of the object detection network, we proposed the PfConv module to improve model performance without increasing the size of the AOD-Net model and introduced the MS-SSIM + $\ell_{2}$ loss function to enhance the generalization of the dehazing model under various haze densities and multiple haze conditions. To tackle the issue of small-object detection, we proposed the CCFF-BiFPN-P2 structure based on RT-DETR, embedding a small-object layer into the BiFPN structure to recover inadvertently filtered critical feature information. To evaluate the performance of DST-DETR, we conducted experiments with PAOD-Net and ST-DETR both independently and jointly, comparing them with several mainstream dehazing models and object detection models. The experimental results indicate that our image restoration and detection performance surpass other algorithms, demonstrating robust performance under different haze densities and multiple haze conditions, as well as excellent detection capabilities in various backgrounds and occlusion scenarios. Furthermore, DST-DETR can be applied to public foggy datasets beyond safety helmet detection and achieve excellent detection performance. These underscore the practical application value of DST-DETR.

Although the designed DST-DETR framework has achieved promising results in helmet detection under foggy conditions, there is room for improvement. For instance, integrating it with object tracking algorithms could enhance site supervision, ensuring the safety of construction workers. Additionally, exploring ways to overcome more complex adverse weather conditions, such as low light, rainy, and snowy days, would be beneficial. Furthermore, applying the proposed framework to other object detection domains, especially in natural foggy scenes without dedicated dehazing datasets, could further enhance the model’s generalization and robustness.

## Figures and Tables

**Table 1:** Quantitative evaluation of the haze test sets for various concentrations and average run times for each model. ↑ indicates that better methods should achieve higher scores for this metric. ↓ indicates that better methods should achieve lower scores for this metric.

	DCP	CAP	DehazeNet	MSCNN	AOD-Net	FFA-Net	Ours
	Light Haze						
PSNR(dB) ↑	16.70	19.71	20.53	17.75	16.04	19.25	19.92
SSIM ↑	0.8603	0.8882	0.9017	0.8581	0.8026	0.8896	0.8794
	Medium Haze						
PSNR(dB) ↑	12.90	14.53	15.67	14.45	14.32	15.87	16.39
SSIM ↑	0.7854	0.8079	0.8297	0.8091	0.7436	0.8254	0.8239
	Heavy Haze						
PSNR(dB) ↑	8.96	9.47	10.37	9.94	12.21	10.67	14.79
SSIM ↑	0.6402	0.6416	0.6886	0.6832	0.6624	0.7060	0.7670
Time(s) ↓	0.19	0.78	3.86	4.26	0.03	1.24	0.03

**Table 2:** Ablation experiments of PAOD-Net under multiple haze levels.

PfConv	MS-SSIM	$\ell_{1}$	$\ell_{2}$	PSNR	SSIM
		✓		15.05	0.7798
			✓	14.19	0.7362
	✓	✓		15.60	0.8029
	✓		✓	15.88	0.8147
✓		✓		15.66	0.7986
✓			✓	16.32	0.7924
✓	✓	✓		16.28	0.7949
✓	✓		✓	17.03	0.8234

**Table 3:** The mAP@0.5 detection results of different models under different haze levels.

Training Set	Test Set	Faster R-CNN	SSD	YOLOv5	YOLOv7	YOLOv8	YOLOv9	YOLOv10	RT-DETR	Ours
Light	Light	0.6746	0.7648	0.9062	0.9301	0.9059	0.9108	0.9070	0.9137	0.9476
Medium	Medium	0.6360	0.7566	0.9002	0.9251	0.9019	0.9067	0.9042	0.9136	0.9469
Heavy	Heavy	0.6247	0.7429	0.8861	0.9171	0.8929	0.8985	0.8950	0.9075	0.9441

**Table 4:** Comparison of object detection results across different models under multiple haze levels.

Training Set	Test Set	Model	Params (M)	FPS	mAP@0.5	mAP@0.5:0.95
Multiple	Multiple	YOLOv5-M	21.2	94	0.8918	0.5564
		YOLOv7	36.9	30	0.9208	0.5464
		YOLOv8-M	25.9	93	0.8944	0.5580
		YOLOv9-M	20.0	68	0.9031	0.5661
		YOLOv10-M	15.4	84	0.8992	0.5559
		RT-DETR-L	32.0	65	0.9073	0.5632
		RT-DETR-R18	19.9	84	0.9054	0.5580
		Ours	22.8	71	0.9481	0.6051

**Table 5:** Ablation experiments of ST-DETR under multiple haze levels.

Training Set	Test Set	ResNet-18	BiFPN	P2	Precision	Recall	AP@0.5 (Helmet)	AP@0.5 (Person)
Multiple	Multiple	✓			0.9152	0.8526	0.9034	0.9074
		✓	✓		0.9252	0.8806	0.9346	0.9383
		✓		✓	0.9195	0.8669	0.9185	0.9206
		✓	✓	✓	0.9343	0.8991	0.9466	0.9496

**Table 6:** Ablation experiments of DST-DETR under multiple haze levels.

Training Set	Test Set	AOD-Net	PAOD-Net	RT-DETR	ST-DETR	mAP@0.5
Multiple	Multiple			✓		0.8864
					✓	0.8971 (+0.107)
		✓		✓		0.8968
		✓			✓	0.9192 (+0.224)
			✓	✓		0.9054
			✓		✓	0.9481 (+0.427)

**Table 7:** Quantitative evaluation of each model’s performance on public datasets.

Dataset	Model	Precision	Recall	mAP@0.5	mAP@0.5:0.95
RTTS	DSNet [1]	-	-	0.5068	-
	IA-YOLO [2]	-	-	0.5003	-
	BAD-Net [4]	-	-	0.5315	-
	RT-DETR	0.7373	0.5850	0.6402	0.3659
	DST-DETR	0.7599	0.6268	0.6838	0.3792
SFID	FINet [3]	0.9936	0.9878	0.9941	0.8816
	RT-DETR	0.9878	0.9833	0.9879	0.8416
	DST-DETR	0.9926	0.9904	0.9945	0.8763

## Data Availability

The data presented in this study are available on request from the corresponding author.
